# Circulatory system disease mortality and occupational exposure to radon progeny in the cohort of Newfoundland Fluorspar Miners between 1950 and 2016

**DOI:** 10.1007/s00420-022-01932-x

**Published:** 2022-11-01

**Authors:** Paul J. Villeneuve, Howard I. Morrison, Karena Volesky, Rachel S. D. Lane

**Affiliations:** 1 Department of Neuroscience, Faculty of Science, Carleton University, Health Sciences Building, Room 6307, 1125 Colonel By Drive, Ottawa, ON K1S 5B6 Canada; 2 Centre for Surveillance and Applied Research, Public Health Agency of Canada, Ottawa, ON Canada; 3 McGill University, Montreal, QC Canada; 4 Canadian Nuclear Safety Commission, Ottawa, Canada

**Keywords:** Radon progeny, Cardiovascular disease, Miner, Cohort study

## Abstract

**Objectives:**

Exposure to ionizing radiation may increase the risk of circulatory diseases, including heart disease. A limited number of cohort studies of underground miners have investigated these associations. We previously reported a positive but non-statistically significant association between radon progeny and heart disease in a cohort of Newfoundland fluorspar miners. In this study, we report updated findings that incorporate 15 additional years of follow-up.

**Methods:**

The cohort included 2050 miners who worked in the fluorspar mines from 1933 to 1978. Statistics Canada linked the personal identifying data of the miners to Canadian mortality data to identify deaths from 1950 to 2016. We used previously derived individual-level estimates of annual radon progeny exposure in working-level months. Cumulative exposure was categorized into quantiles. We estimated relative risks and their 95% confidence intervals using Poisson regression for deaths from circulatory, ischemic heart disease and acute myocardial infarction. Relative risks were adjusted for attained age, calendar year, and the average number of cigarettes smoked daily.

**Results:**

Relative to the Newfoundland male population, the standardized mortality ratio for circulatory disease in this cohort was 0.82 (95% CI 0.74–0.91). Those in the highest quantile of cumulative radon progeny exposure had a relative risk of circulatory disease mortality of 1.03 (95% CI 0.76–1.40) compared to those in the lowest quantile. The corresponding estimates for ischemic disease and acute myocardial infarction were 0.99 (95% CI 0.66–1.48), and 1.39 (95% CI 0.84–2.30), respectively.

**Conclusions:**

Our findings do not support the hypothesis that occupational exposure to radon progeny increases the risk of circulatory disease.

## Introduction

The possibility that exposure to ionizing radiation increases the risk of circulatory system disease at low and moderate doses has important implications given the widespread and increasing use of medical applications such as computed topography scans, nuclear medicine, and radiotherapy (Mettler 2019). Understanding these health risks is also important given the relatively large numbers of workers exposed to ionizing radiation. In Canada, for example, approximately 174,000 individuals were exposed to ionizing radiation in occupational settings in 2018 (Health Canada 2021). Our knowledge of the health risks of chronic exposure to ionizing radiation has been informed largely on risks observed at much higher doses, and usually shorter intervals, such as those exposed from underground mining, industrial accidents, or atomic bomb survivors (National Research Council 2006). A recent analysis of 66 countries concluded that 16.5% of lung cancers are attributable to exposure to residential radon (United Nations Scientific Committee on the Effects of Atomic Radiation 2020). As radon represents approximately 42% of the natural source of radiation in Canada (Government of Canada 2011), if exposure to radon were to increase the risk of circulatory system disease the population health impacts would be substantial. Heart disease is currently the second leading cause of death among Canadians, and an estimated 2.4 million Canadians live with ischemic heart disease (Government of Canada 2017).

Findings from epidemiological investigations across a diverse series of study populations provide compelling evidence that ionizing radiation causes circulatory disease. This evidence includes higher rates of heart disease following radiotherapy treatment (> 30 Gy) for breast cancer (Darby et al. 2013), and Hodgkin’s lymphoma (Darby et al. 2010), as well as experimental animal studies that found ionizing radiation accelerated the development of atherosclerotic lesions (Stewart et al. 2006). In addition, long-term follow-up studies of the Japanese atomic bomb (A-bomb) survivors Life Span Study (LSS) have also observed increased circulatory disease mortality risks for radiation doses that exceeded 0.5 Gy (Preston et al. 2003). In contrast, Preston and colleagues found no direct evidence of radiation effects on heart disease and stroke for doses less than about 0.5 Sv using the 1950–1997 mortality follow-up of the LSS (Preston et al. 2012). Elsewhere, Schollnberger and colleagues found statistically significant increases in radiation risk for heart disease and cerebrovascular diseases at higher doses (Schollnberger et al. 2018). However, there are uncertainties about radiation risks below 0.75 Gy for cerebrovascular diseases and 2.6 Gy for heart diseases (Schollnberger et al. 2018). A detailed synthesis of the epidemiology literature on ionizing radiation and circulatory diseased was recently published by Little et al. (2021). They found strong evidence of a statistically significant excess risk of the major types of circulatory disease, specifically ischemic heart disease and stroke, in high, moderate- or low-dose exposed groups. However, the patterns of risk reported were not straightforward, with differences between high-dose medical studies and moderate/low doses in the Japanese atomic bomb survivors and various occupational and environmental studies. They also found that the risk per unit dose varied across different dose rates and fractionated exposures, as well uncertainties related to the possible confounding and modifying effects of well-known risk factors that were often not considered in published studies.

Studies of miners have provided important insights into the relationship between radon and cancer (especially lung cancer); however, relatively few have assessed the dose–response relationship between radon and circulatory diseases. Most of this research has relied on comparisons of circulatory disease death rates among miners to the general population (Little et al. 2021; McGale and Darby 2005). However, studies using only standardized mortality ratios (SMRs) can be biased by the health worker effect (Choi 1992) which arises because those employed in physically demanding jobs tend to be healthier than the general population. A few studies have characterized the radiation risk of circulatory diseases using internal cohort comparisons (Drubay et al. 2015; Kreuzer et al. 2010; Lane et al. 2010; Villeneuve et al. 2007a; Xuan et al. 1993). In the largest of these studies, the German cohort of uranium miners, no associations were found between cumulative exposure to radon and either heart disease or cerebrovascular disease (Kreuzer et al. 2010). Another analysis of approximately five thousand French uranium miners found that cumulative exposure to radon was positively associated with the risk of cerebrovascular disease with an excess relative risk (ERR) per 100 WLM of 0.41 (95% CI 0.04–1.03) (Rage et al. 2015). A nested case–control analyses within this same cohort found that cumulative exposure was associated with an increased risk of circulatory disease mortality (Drubay et al. 2015). While studies of uranium workers provide valuable insights—these workers are exposed to both external gamma rays and internal radiation (radon, long-lived radionuclides).

The source of radon progeny exposure among the fluorspar miners was via the groundwater that ran through the mines. As a result, exposure–response analyses of radon and health outcomes in this cohort are not confounded by other sources of radiation, however, these workers were exposed to other substances such as diesel and silica that are recognized carcinogens and that increase the risk of cardiovascular disease. We have previously analyzed associations between radon and circulatory disease mortality in this cohort The earlier paper included mortality follow-up from 1950 to 1990 and found elevated, but not statistically significant, relative risks for coronary heart disease (CHD) mortality among miners with a cumulative radon exposure exceeding 1,000 WLMs (RR = 1.5, 95% CI 0.77–2.75) (Villeneuve and Morrison 1997). The more recently published analysis included follow-up to the end of 2001, also found a modestly elevated risk of deaths from coronary heart disease, and acute myocardial infarction (Villeneuve et al. 2007a). However, no clear exposure–response trends were found, and the associations were not statistically significant.

Our objective is to conduct an updated analysis of the relationship between radon exposure and circulatory disease in the cohort of Newfoundland fluorspar miners, with an additional 15 years of mortality follow-up (1950–2016) since our previous analysis. The additional follow-up provides the increased statistical power to assess these associations in a cohort that is relatively small (~ 2000 miners). In this paper, we use the term “radon” throughout, and this refers to radon (^222^Rn) and its short-lived progeny (^218^Po, ^214^Pb, ^214^Bi, and ^214^Po), synonymously.

## Methods

### Study population

A detailed description of the cohort of Newfoundland Fluorspar miners has previously been published (Morrison et al. 1998; Villeneuve et al. 2007a, b). Briefly, the all-male cohort initially included 2661 miners, employed between 1933 (date that commercial mining started in St. Lawrence) to 1978 (closure of the last mine) by either the St. Lawrence Fluorspar Company or Newfoundland Fluorspar Limited. During their employment, 2111 miners worked underground, where they were exposed to radon via groundwater seepage, and 550 remaining employees worked exclusively on the surface. Each employee’s annual work history was constructed from employment records.

Employees lacking the personal identifying data required for record linkage to Canada’s national mortality databases were excluded. Most of these workers were employed for a short time during World War II. Miners who died prior to the start of mortality follow-up in 1950, and 19 miners born before 1900 for whom no matching death record was found were excluded from analyses. These miners were either lost to follow-up or died before 1950 when death registration in the Canadian Mortality Database was incomplete. Newfoundland was the last province to join the Canada confederation on 31 March 1949.

### Radon

For each underground miner, an annual measure of exposure to radon was derived by combining sampling measures with information on mine location and architecture, amount of water inflow, miners’ working hours, general working conditions, and place of residence (1933–1960). Those who worked on the surface were assumed to have only background levels of radon exposure.

The method of radon sampling differed from the start of commercial mining in 1933 to the last mine’s closure in 1978. Prior to 1960, radon levels were not monitored, rather 80 samples were taken from 50 underground locations in the principal mine—the radon concentrations of these samples ranged from 0.4 to 193 working levels (WLs). To assign pre-1960 exposures, Corkill and Dory performed a retrospectively reconstructed estimated doses using data from the 80 samples supplemented with information collected from inspector reports, Commission hearings and miners’ themselves (Corkill and Dory 1984).

In 1960, mechanical ventilation was installed in the mines. Shortly thereafter, regular monitoring of radon levels began. Between 1961 and 1967, between 400 and 700 samples were taken annually to measure radon progeny in the mines (Morrison et al. 1998). Mean annual radon exposure was 64.8 WLMs pre-ventilation (1955–1959) for underground miners; in comparison, it was 1.3 WLMs from 1961 to 1965.

Later, from 1968 to 1978, radon levels were measured daily for each miner at the location in the mine they worked. For underground miners, the mean exposure from 1968 to 1978 decreased to 0.6 WLM.

### Cigarette smoking

A series of smoking surveys were administered to miners in 1966, 1970, and 1978 (Morrison et al. 1988). We were involved with additional surveys that collected smoking behaviour data in 1993 and 2003. For the more recent surveys, local interviewers in St. Lawrence Newfoundland were hired, and in 2003, internet search engines were also used to help locate eligible participants. Data from the five surveys were combined, and miners were categorized according to their last known smoking status (never or ever). The average number of cigarettes smoked daily was derived using data collected across all surveys.

### Mortality data

A linked file that contained occupational data and mortality status from the 2007 mortality update (Villeneuve et al. 2007a, b), stored at the Canadian Nuclear Safety Commission, contained 2121 observations—one for each miner. This file included personal identifying data (given name, surname, birth date), annual radon exposure, vital status, and the smoking survey data. We provided this file to Statistics Canada to update the mortality follow-up between 1950 and 31 December 2016. This linkage used the Canadian Mortality Data Base (from 1950 to 2011) and the Canadian Vital Statistics Death Database (CVSD; from 1930 to 1949 and 2011 onwards). The CVSD is an annual administrative survey collecting demographic and the fact of death data from each provincial/territorial vital statistic registry on all deaths occurring in Canada (Statistics Canada 2019). If a link could not be established, miners were assumed to be alive at the end of follow-up. Links utilized probabilistic and deterministic methods, via the ‘matchit’ program in Stata 14.2. After identifying deaths from the linkage, PJV performed a manual review of all death links. Due to the lack of completeness of the CVSD before 1950, and as an only fact of death and not underlying cause of death was available, our analyses are based on mortality outcomes from 1950 onwards. This is the same approach used in previous analyses of this cohort (Morrison et al. 1985; Morrison and Villeneuve 1995; Villeneuve and Morrison 1997; Villeneuve et al. 2007b).

We classified the underlying cause of death for each miner according to the International Classification of Disease 9th (prior to 2000) and 10th revisions (2000 onwards). For this paper, deaths were categorized as described in Table 1. We also considered investigating the relationship between radon exposure and hypertension however, the relatively small numbers of death (~ 10) precluded exposure–response modeling.

**Table 1:** Number of deaths (1950–2016) for selected circulatory diseases among the cohort of Newfoundland fluorspar miners

Underlying cause of death	ICD-9 codes	ICD-10 codes	Deaths^a^ (rounded)
Circulatory diseases	390–459	I00–I99	480
Ischemic heart disease	410–414	I20–I25	290
Acute myocardial infarction	410	I21–I22	175
Stroke (cerebrovascular disease)	430–438	I63	60
Hypertension	401–405	I10	10

*ICD* International classification of diseases

The number of deaths were rounded to the base unit of 5 as per Statistics Canada’s rules of disclosure governing the use of files linked to national mortality data

### Statistical analysis

We first described the cohort in terms of key characteristics: type of miner (surface or underground), year of death, year of birth, lifetime smoking status, and age at first exposure to radon. We classified the data by categories of attained age (5-year age categories), and calendar period (10-year categories). The software program Epicure was used to tabulate the person-years of follow-up.

We used Poisson regression to perform internal cohort comparisons to describe the risk of mortality in relation to cumulative radon exposure. We did undertake a comparison of the mortality of the cohort to the general population as circulatory deaths, however, it is important to recognize that the standardized mortality ratio is prone to the healthy worker effect bias. The program EPICURE was used to calculate the person-years of follow-up and to fit the Poisson regression models (Preston 2015). As in previous analyses, we applied a latency period of 5 years when we modelled cumulative radon exposure. We assessed the risk of circulatory and ischemic disease mortality in relation to cumulative radon exposure by using a quintile-based approach based on the number of observed circulatory deaths. We calculated relative risks (RR) and their corresponding 95% confidence intervals (CIs) for four of the five quantiles of cumulative radon exposure (measured in WLM) with the lowest category serving as the referent. We adjusted these risks for attained age, calendar period, and smoking status. A linear excess relative risk (ERR) model was also fit, and this took the functional form:

$$\mathrm{ERR}=1+\beta \mathrm{WLM}.$$

This excess relative risk was adjusted for attained age, calendar period and average number of cigarettes smoked daily. We categorized the number of cigarettes smoked daily by using tertile–based cut-points (< 15, 15–21.7, > 21.7) such that the number of deaths from the circulatory disease were approximately equal. This is done to optimize the standard errors of the derived risk estimates for smoking. We repeated these analyses after excluding those with no smoking data to assess possible selection bias.

All presented frequencies, including counts, deaths and person-years, were rounded to the base unit five to adhere to Statistics Canada’s rules of disclosure governing the use of records linked to national mortality data. However, all relative risks were estimated using the actual (not rounded) mortality counts. We performed all analyses at Statistics Canada’s Research Data Center Network.

### Funding and ethics

This study was funded by the Canadian Nuclear Safety Commission. Ethical approval was received by Carleton University’s Research Ethics Board (Clearance #: 108564). As part of its record linkage process, Statistics Canada reviewed and approved the study.

## Disclosure

The authors have no disclosures to report.

## Results

In total, the cohort included 315 miners who worked exclusively on the surface, while 1735 miners worked underground (Table 2). From 1950 to 2016, our study had 81,650 person-years of mortality follow-up, with 67% of miners deemed dead and 690 miners deemed to still be alive based on the record linkage. Among 2055 miners, 1080 (52.6%) miners had some cigarette smoking data, and of these 85% had ever smoked.

**Table 2:** Descriptive characteristics of the cohort of Newfoundland fluorspar miners with mortality follow-up from 1950 to 2016

Characteristics	Number of miners	%
Surface miner (exclusively)	315	15.3
Underground miner	1735	84.7
Age at first exposure < 20 year	375	18.2
Age at first exposure 20–24 year	535	26.0
Age at first exposure 25–29 year	290	14.1
Age at first exposure 30–39 year	320	15.6
Age at first exposure 40 + year	215	10.5
Birth year		
Before 1900	175	8.5
1900–1909	170	8.3
1910–1919	390	19.0
1920–1939	420	20.4
1940–1949	340	16.5
1950–1959	475	23.1
Death year		
1935–1949	25	1.2
1950–1959	90	4.4
1960–1969	185	9.0
1970–1979	230	11.2
1980–1989	230	11.2
1990–1999	240	11.7
2000–2009	330	16.1
2010–2016	135	6.7
Alive at end of follow-up	690	33.6
First employment		
1933–1939	285	13.9
1940–1944	590	28.6
1945–1949	115	5.6
1950–1954	190	9.3
1955–1959	80	4.0
1960–1964	240	11.6
1965–1969	375	18.2
1970–1976	180	8.8
Lifetime smoking status		
Never	160	14.5
Ever	940	85.5
Unknown	955	
Cigarettes smoked daily		
Never smoker	160	7.8
< 10	45	2.2
10–19	270	13.1
20–29	420	20.4
30–39	120	5.9
40 +	65	3.2
Unknown	970	47.2

The number of miners were rounded to the base unit of 5 as per Statistics Canada’s rules of disclosure governing the use of files linked to national mortality data

The relative risks of mortality for selected circulatory causes of death according to the average number of cigarettes smoked daily are presented in Table 3. The risk of circulatory disease mortality among those who smoked more than 21.7 cigarettes per day relative to those smoking less than 15 cigarettes a day was 1.55 (95% CI 1.10–2.19). Increased risks from smoking were also observed for ischemic heart disease mortality and myocardial infarction. Due to the small numbers of death from cerebrovascular disease, we were unable to present these smoking risk estimates.

**Table 3:** Relative risk of selected circulatory disease mortality (1950–2016) by the average number of cigarettes smoked daily among the cohort of Newfoundland fluorspar miners

Cigarettes smoked daily	Deaths	Person-years of follow-up	Relative Risk* (95% CI)		Relative Risk*† (95% CI)	
Circulatory disease mortality						
< 15	50	13,520	1.0		1.0	
15– < 21.7	75	16,933	1.09	0.76–1.56	1.12	0.79–1.60
21.7 +	85	14,900	1.55	1.10–2.19	1.61	1.13–2.27
Unknown	265	36,300	1.16	0.86–1.58		
Ischemic heart disease						
< 15	30	13,520	1.0		1.0	
15– < 21.7	45	16,933	0.99	0.63–1.57	1.02	0.65–1.62
21.7+	60	14,900	1.67	1.09–2.57	1.71	1.11–2.64
Unknown	150	36,300	1.14	0.78–1.68		
Acute myocardial infarction						
< 15	20	13,520	1.0		1.0	
15– < 21.7	25	16,933	0.88	0.49–1.59	0.90	0.50–1.53
21.7+	35	14,900	1.61	0.93–2.78	1.62	0.94–2.79
Unknown	90	36,300	1.05	0.64–1.71		

*Relative risks were adjusted by attained age, calendar period, and average number of cigarettes smoked daily

^†^Miners with missing smoking data were excluded

The relative risks of cause-specific circulatory disease mortality across quintiles of cumulative radon exposure are shown in Table 4. Similar risk patterns were observed among the restricted cohort that had smoking data available, however, the confidence intervals of these estimates were larger. The linear excess relative risks (ERR) per 100 WLM for all circulatory diseases, ischemic heart disease, acute myocardial infarction and cerebrovascular mortality were: 0.002 (95% CI − 0.020 to 0.023), 0.005 (95% CI − 0.031 to 0.022), 0.026 (95% CI − 0.018 to 0.070) and − 0.35 (95% CI − 0.76 to 0.00) [data not shown]. None of these estimates attained statistical significance (at *p* < 0.05). The relative risk of circulatory disease mortality in the highest quintile relative to the lowest was 1.03 (95% CI 0.76–1.40). An increased risk was observed in the highest quintile for an acute myocardial infraction, while a lower risk was observed for cerebrovascular disease (Table 4). However, neither was statistically significant, and there was no statistically significant linear trend observed between any of the four circulatory causes of death and radon in the cohort.

**Table 4:** Relative risk of selected circulatory disease mortality (1950–2016) by cumulative radon exposure among the cohort of Newfoundland fluorspar miners

Cumulative exposure to radon in WLM (quantiles)	Deaths	Person-years of follow-up	Relative Risk* (95% CI)		Relative Risk (95% CI)*†				
Circulatory disease mortality									
0	105	17,510	1.0			1.0			
> 0– < 4.6	90	24,315	0.99	0.73–1.34		1.05		0.55–1.99	
4.6– < 25.9	95	16,715	1.00	0.75–1.34		1.23		0.66–2.02	
25.9– < 94.7	95	11,400	1.10	0.82–1.47		1.21		0.70–2.14	
94.7+	95	11,710	1.03	0.76–1.40		1.06		0.61–1.85	
Ischemic heart disease									
0	55	17,510	1.0			1.0			
> 0– < 4.6	55	24,315	0.98	0.66–1.45		1.24		0.55–2.80	
4.6– < 25.9	60	16,715	1.06	0.73–1.56		1.31		0.63–2.72	
25.9– < 94.7	60	11,400	1.19	0.82–1.74		1.31		0.62–2.77	
94.7+	55	11,710	0.99	0.66–1.48		1.14		0.55–2.37	
Acute myocardial infarction									
0	30	17,510	1.0			1.0			
> 0– < 4.6	30	24,315	1.08	0.64–1.82		1.30		0.42–3.98	
4.6– < 25.9	30	16,715	1.01	0.60–1.70		1.26		0.47–3.41	
25.9– < 94.7	40	11,400	1.36	0.83–2.23		1.13		0.41–3.12	
94.7+	40	11,710	1.39	0.84–2.30		1.56		0.60–4.06	
Cerebrovascular disease									
0	10	17,510	1.0						
> 0– < 4.6	10	24,315	0.81	0.33–2.01			RRs suppressed		
4.6– < 25.9	15	16,715	1.56	0.72–3.40			Due to small		
25.9– < 94.7	15	11,400	1.26	0.56–2.85			Counts		
94.7+	10	11,710	0.69	0.26–1.86					

*Relative risks were adjusted by attained age, calendar period, and average number of cigarettes smoked daily

^†^Miners with missing smoking data were excluded

## Discussion

This paper presents updated risk estimates for occupational radon exposure and circulatory disease in the Newfoundland fluorspar miners’ cohort. These analyses extend previous analyses of the same outcome and now include over 65 years of mortality follow-up overall, and includes internal cohort analyses for myocardial infarction and cerebrovascular disease. There were no statistically significant associations between cumulative exposure to radon and any of the four circulatory disease causes of death studied. Indeed, the relative risk of mortality for all circulatory deaths and ischemic heart disease (among those in the highest quintile of exposure compared to the lowest) were null. The findings are consistent with previously published analyses of this cohort (Villeneuve et al. 2007a; Villeneuve and Morrison 1997) and cohorts of uranium miners (Drubay et al. 2015; Kreuzer et al. 2006).

We pursued these updated analyses due to the availability of an additional 15 years of follow-up, which was anticipated to increase our statistical power. The number of observed circulatory disease deaths increased by 22% (from 389 to 475) with the longer follow-up. While the number of circulatory deaths is much less than the number in the German uranium cohort (475 vs 5417), it is more than the number of deaths examined via internal cohort analysis in the French uranium cohort (475 versus 76). Despite the increased number of deaths, the general patterns of risk for each cause of death did not change substantially. It is our view that further extension of the mortality follow-up of the cohort will not yield any further insights on possible associations between radon and circulatory diseases in this cohort. Our findings do not support the hypothesis that exposure to radon increases the risk of mortality from these causes.

As with previous analyses of this cohort, we excluded miners who died before 1950. This was done because national death registrations were incomplete before this time. It is also important to note that Newfoundland did not join Canada until 1949. The decision to exclude these workers, who would have had higher exposure to radon could introduce some selection bias. However, it is our view that this potential selection bias would not have changed our findings in a meaningful way. First, relative to the total number of deaths in the cohort during the nearly 70 years of follow-up time, the number of deaths before 1950 would have been small given that: mining operations began in the early 1930s, and the miners would have had to be young and healthy enough to work. Moreover, while Statistics Canada did identify 24 deaths before 1950, only four of these deaths were from circulatory disease, and three of these miners had accrued no cumulative exposure to radon. As a whole though, the cumulative exposure to radon among these 24 miners was nearly double the exposure (636 vs 318 WLM) relative to those who were followed from 1950 onwards.

Unlike many other studies of underground miners, we were able to adjust for the possible confounding role of cigarette smoking. As expected, smoking was positively related to the four circulatory diseases under study. While we were able to adjust for smoking, attained age, and calendar period effects, we recognize that a limitation of this work is that we are unable to adjust for other cardiovascular disease risk factors including hypercholesterolemia, diabetes, hypertension, or obesity—these risk factor data were not collected, and conditions such as diabetes and hypertension are not reliably captured on death certificates. The four aforementioned risk factors, along with cigarette smoking, are estimated to be responsible more than half of cardiovascular deaths in the United States (Patel et al. 2015).

As done with prior studies, including those examining lung cancer outcomes, a five-year lag was incorporated into the modelling (Kreuzer et al. 2006; Villeneuve et al. 2007b). This lag assumes that radon exposures occurring in the previous five years are not related to circulatory system disease mortality. While different lags were not utilized as sensitivity analyses here, the last analysis of this cohort examined zero and 10 year lags and found that they did not alter the findings (Villeneuve et al. 2007a).

The characterization of occupational exposure to radon in this cohort is subject to exposure measurement error. The magnitude of this error is larger shortly after the mines began operation in the 1930s. The estimates of exposure during this early period were based on a back-extrapolation of measured concentrations, knowledge of the architecture of the mine, and well as interviews to understand the working conditions at the time. We categorized miners into quintiles of cumulative exposure to minimize the impacts of this measurement error.

## Conclusions

In summary, this study confirms findings from the previous analysis of this cohort indicating that chronic low-level doses of radon do not statistically significantly elevate the risk of circulatory system disease. This work adds to the body of evidence on the health effects of occupational radiation exposure to support radiation protection regulations.
