# Correction: Circulatory system disease mortality and occupational exposure to radon progeny in the cohort of Newfoundland Fluorspar Miners between 1950 and 2016

**DOI:** 10.1007/s00420-022-01945-6

**Published:** 2022-12-14

**Authors:** Paul J. Villeneuve, Howard I. Morrison, Karena Volesky, Rachel S. D. Lane

**Affiliations:** 1 Department of Neuroscience, Faculty of Science, Carleton University, Health Sciences Building, Room 6307, 1125 Colonel By Drive, Ottawa, ON K1S 5B6 Canada; 2 Centre for Surveillance and Applied Research, Public Health Agency of Canada, Ottawa, ON Canada; 3 McGill University, Montreal, QC Canada; 4 Canadian Nuclear Safety Commission, Ottawa, Canada

**Correction: International Archives of Occupational and Environmental Health** 10.1007/s00420-022-01932-x

The article “Circulatory system disease mortality and occupational exposure to radon progeny in the cohort of Newfoundland Fluorspar Miners between 1950 and 2016”, written by Paul J. Villeneuve, Howard I. Morrison, Karena Volesky and Rachel S. D. Lane was originally published electronically on the publisher’s internet portal on 01 November 2022 without open access. With the author(s)’ decision to opt for Open Choice the copyright of the article changed on 29 November 2022 to © The Author(s) 2022 and the article is forthwith distributed under a Creative Commons Attribution 4.0 International License, which permits use, sharing, adaptation, distribution and reproduction in any medium or format, as long as you give appropriate credit to the original author(s) and the source, provide a link to the Creative Commons licence, and indicate if changes were made. The images or other third party material in this article are included in the article’s Creative Commons licence, unless indicated otherwise in a credit line to the material. If material is not included in the article’s Creative Commons licence and your intended use is not permitted by statutory regulation or exceeds the permitted use, you will need to obtain permission directly from the copyright holder. To view a copy of this licence, visit http://creativecommons.org/licenses/by/4.0.

